# Aged *PROP1* Deficient Dwarf Mice Maintain ACTH Production

**DOI:** 10.1371/journal.pone.0028355

**Published:** 2011-12-01

**Authors:** Igor O. Nasonkin, Robert D. Ward, David L. Bavers, Felix Beuschlein, Amanda H. Mortensen, Catherine E. Keegan, Gary D. Hammer, Sally A. Camper

**Affiliations:** 1 Department of Human Genetics, University of Michigan, Ann Arbor, Michigan, United States of America; 2 Graduate Program in Cellular and Molecular Biology, University of Michigan, Ann Arbor, Michigan, United States of America; 3 Department of Internal Medicine, University of Michigan Medical School, Ann Arbor, Michigan, United States of America; 4 Endocrine Research Unit, University Clinic Munich, Munich, Germany; 5 Department of Pediatrics and Communicable Diseases, University of Michigan Medical School, Ann Arbor, Michigan, United States of America; Catholic University Medical School, Italy

## Abstract

Humans with *PROP1* mutations have multiple pituitary hormone deficiencies (MPHD) that typically advance from growth insufficiency diagnosed in infancy to include more severe growth hormone (GH) deficiency and progressive reduction in other anterior pituitary hormones, eventually including adrenocorticotropic hormone (ACTH) deficiency and hypocortisolism. Congenital deficiencies of GH, prolactin, and thyroid stimulating hormone have been reported in the *Prop1^null^* (*Prop1^-/-^*) and the Ames dwarf (*Prop1^df/df^*) mouse models, but corticotroph and pituitary adrenal axis function have not been thoroughly investigated. Here we report that the C57BL6 background sensitizes mutants to a wasting phenotype that causes approximately one third to die precipitously between weaning and adulthood, while remaining homozygotes live with no signs of illness. The wasting phenotype is associated with severe hypoglycemia. Circulating ACTH and corticosterone levels are elevated in juvenile and aged *Prop1* mutants, indicating activation of the pituitary-adrenal axis. Despite this, young adult *Prop1* deficient mice are capable of responding to restraint stress with further elevation of ACTH and corticosterone. Low blood glucose, an expected side effect of GH deficiency, is likely responsible for the elevated corticosterone level. These studies suggest that the mouse model differs from the human patients who display progressive hormone loss and hypocortisolism.

## Introduction

Congenital pituitary hormone deficiency in humans occurs with a frequency of approximately 1 in 4000 live births and is caused primarily by mutations in genes important for pituitary development [1], [2]. Multiple pituitary hormone deficiency (MPHD) results from a variety of transcription factor mutations, including mutations in *PROP1*, *POU1F1 (PIT1)*, *HESX1*, *LHX3, LHX4, OTX2, SOX2, SOX3,* and *GLI2* (reviewed in [3]). Mutations in *POU1F1* almost always cause deficiencies in GH, prolactin (PRL), and thyroid stimulating hormone (TSH) in addition to overall pituitary hypoplasia [4], [5], [6], [7]. Mutations in *Prophet of PIT1 (PROP1*) are the most common known causes of MPHD in humans. The hormone deficiencies are similar to those caused by *POU1F1* mutations, except that the deficiencies include reduced gonadotropin production requiring sex hormone substitution and there is a strong tendency toward progressive hormone loss leading to lower circulating adrenocorticotropic hormone (ACTH) later in life, requiring glucocorticoid replacement therapy [8], [9], [10], [11], [12], [13]. Another interesting difference between *PROP1* and *POU1F1* patients is the tendency of patients with *PROP1* mutations to undergo apparent degeneration of the pituitary gland during childhood [14], [15]. Initially, magnetic resonance imaging analysis may reveal a hyperplastic, or enlarged, pituitary gland, which usually evolves to a hypoplastic appearance a year or so later. The progressive hormone loss and transient pituitary hyperplasia associated with *PROP1* mutations are not well understood.

Several mouse models have been used to dissect the mechanism of *Prop1* action in pituitary development and function. The Ames dwarf (*Prop1^df/df^*) and the *Prop1^null^* (*Prop1^-/-^*) mouse mutants recapitulate the human MPHD phenotype in that adult mutants are profoundly deficient in TSH, GH, PRL, have low circulating gonadotropins, and pituitary hypoplasia [16], [17], [18], [19]. Studies in *Prop1* mutant mice show that precursor cells fail to colonize the anterior lobe resulting in reduced cell proliferation and enhanced apoptosis after birth leading to hypoplasia that becomes evident in the weeks after birth [20], [21]. *Prop1* mouse mutants differ from humans with *PROP1* mutations in that the hormone deficits are consistently congenital rather than progressive, thyroid hormone and growth hormone replacement are sufficient for fertility, and there is no clear evidence for transient pituitary hyperplasia.

The genetic background exerts a considerable influence on the phenotype of the *Prop1* deficient mice, although both alleles, *Prop1^df/df^* and *Prop1^-/-^*, have the same features when normalized for genetic background [19]. Similarly, humans with the same mutation in *PROP1* can have different clinical presentations [10]. Enrichment of the 129S1/SvImJ (129) background enhances the frequency with which newborn *Prop1* mouse mutants die of respiratory distress. The lack of pituitary TSH results in fetal hypothyroidism, reduced expression of the thyroid hormone inducible transcription factor TTF1 in the lung, and inadequate production of surfactants, known target genes of TTF1. The lungs fail to inflate, causing respiratory distress and lethality [19]. Increasing the contribution of C57BL/6J (B6) strain background tended to protect against this survival defect in newborns. Here we report that the B6 background increases the sensitivity of *Prop1* deficient mice to lethality after weaning. The reason for this juvenile lethality has not been explored.

Corticotroph development does not appear to be affected in the *Prop1* deficient mice, and corticosterone levels are not reduced in newborn mutants [19], [22], [23]. Because most *PROP1* patients who have been closely followed appear to have evolving hypocortisolism [13], and the underlying cause of the juvenile lethality of *Prop1* mutant mice is not known, it is necessary to investigate pituitary adrenal function in young and old *Prop1* deficient mice on a sensitized (B6) genetic background.

We report no evidence for progressive ACTH loss in juvenile and young adult *Prop1* deficient mice. In contrast, our results show increased serum ACTH and corticosterone levels in young and old *Prop1* mutants. The pituitary-adrenal axis is functional in young adult *Prop1^null^* mice as demonstrated by elevated activity in response to restraint stress. *Prop1* mutants have significantly reduced blood glucose levels, as expected for GH deficient animals, which could trigger the activation of the pituitary-adrenal axis. Untreated hypoglycemia can cause mortality in both humans and mice [24]. We conclude that both of the *Prop1* mouse alleles we tested on various genetic backgrounds differ from human patients by maintaining elevated pituitary adrenal axis activity through 1 year of age, with no evidence for evolving hypocortisolism.

## Materials and Methods

### Mice

All mice were housed in a 12-h light, 12-h dark cycle with unlimited access to tap water and Purina 5008 or 5020 chows. All procedures using mice were approved by the University of Michigan Committee on Use and Care of Animals, and all experiments were conducted in accordance with the principles and procedures outlined in the NIH Guidelines of the Care and Use of Experimental Animals.

The *Prop1^Sactm1^* heterozygous null mice, referred to here as *Prop1* ^+/−^, were generated from R1 (129/Sv x 129/Sv-CP) ES cells by replacing the coding region of exon 1, intron 1, and a portion of exon 2 with cassettes encoding β-galactosidase and neomycin resistance (19, 37). The chimeras were mated to C57BL/6J mice (B6) (The Jackson Laboratories, Bar Harbor, ME) to generate F1 heterozygous animals and were first analyzed on a mixed F2 C57BL/6J-129S1/SvImJ background (B6/129). The F2 *Prop1^+/^* ^−^ heterozygous mice were backcrossed to B6 mice for four generations to establish the *Prop1^+/^* ^−^ N4 B6 breeding colony, which is theoretically 93.75% pure B6. Mice used in the study of pituitary-adrenal function were from the N4 B6 genetic background unless expressly stated otherwise. *Prop1^-/-^* mice were determined by PCR as previously described [19], [20].

The DF/B-*Prop1^+/df^* stock is not inbred. It was obtained from Dr. A. Bartke at Southern Illinois University in 1988 and maintained at University of Michigan. This stock was backcrossed to B6 to N4 [19].

### Restraint stress and blood collection

Young adult mice (8–10 weeks old, N4 B6) were housed individually 12 hours prior the experiment, with special precautions to avoid stress associated with noise and cage handling. The blood samples were collected in the morning (between 9:00am and 10:30am) by retro orbital bleeding in heparinized collection tubes (Microvette CB300; Sarstedt, Inc., Newton, NC). The retro orbital bleeding was done in less than one minute after initial mouse handling to prevent stress-induced corticosterone release. Animals were subjected to restraint stress for 30 minutes, after which another blood sampling was performed by the same method [25], [26], [27]. Plasma was prepared according to the manufacturer's protocol for the Microvette CB300 (Sarstedt).

For ACTH measurements in non-stressed conditions, animals of various ages were anesthetized with metaphane, rapidly decapitated within less than 1 min from the time of initial handling, and blood samples collected.

### Corticosterone, ACTH, and glucose measurements

ACTH and corticosterone were measured by radioimmunoassay (RIA) in plasma using a ^125^I RIA kit (ICN Diagnostics, Costa Mesa, CA) according to the manufacturer's protocol [28]. The blood-glucose measurements were done using a FreeStyle glucose meter (TheraSense, Alameda, CA). Duplicate measurements were done for each sample collected. According to manufacturer's instructions, glucose levels below 60 mg/dL are considered evidence of hypoglycemia. Glucose measurements were performed on 3.5 to 5 week, 5 to 6.5 week, and 8 to 10 week pre- and post-stressed N4 B6 animals. The device's lowest sensitivity level is 20 mg/dL (http://www.abbottdiabetescare.com). If glucose levels were below the level of detection, an arbitrary number of 19 mg/dL was assigned for the purpose of statistical analysis.

### Histology and Immunohistochemistry

Adrenals were collected immediately after euthanizing and rinsed in ice-cold PBS prior to 1 h fixation in 4% paraformaldehyde on ice (diluted in PBS, pH 7.2). Samples were washed in PBS, dehydrated in a graded series of ethanol, and embedded in paraffin. Seven-micrometer sections were prepared and either stained with hematoxylin and eosin. The 20α-hydroxysteroid dehydrogenase antibody was generously provided by Yacob Weinstein and used at 1:2000-3000 dilution [29].

### Statistical analysis

Data were processed and plotted using StatView software (Abacus Concepts, Inc., Edinburgh, United Kingdom), with the exception of the qRT-PCR data that was processed using Microsoft Excel Software. ANOVA (analysis of variance) and Fisher's exact test were used to evaluate the data. All data are shown as +/− 1 SEM (standard error of the mean). P-values of less than 0.05 were considered to be statistically significant.

## Results

### *Prop1* deficiency can cause postnatal lethality

We analyzed the viability of two different mutant alleles of *Prop1* on several genetic backgrounds. The Ames dwarf mutant, *Prop1^df/df^,* arose spontaneously on a poorly defined genetic background (DF/B), and it carries a missense mutation in the homeodomain, Ser83Pro [18], [30]. We generated a null allele, *Prop1^+/^* ^−^, on a mixed background comprised of C57BL/6J (B6) and 129S1/SvImJ (129) [19]. We frequently observed a crisis in mutant viability after weaning. On the 129/B6 mixed background 37% (13/35) of the *Prop1* ^-/-^ animals exhibited lethargy, wasting, and death between 3 and 7 weeks of age. Death usually occurred within 3-5 days of initial signs of distress. More males were affected than females (p = 0.03). A similar wasting and lethality phenotype was also observed in 27% (6/22) of compound heterozygotes, *Prop1* ^df/-^, on a mixed background.

We back-crossed both strains, DF/B-*Prop1* ^df/+^ and 129/B6*-Prop1* ^+/−^, four times to B6 to be able to compare the phenotypes of the two alleles on a consistent genetic background. We observed identical viability of the homozygous mutants for each allele at two weeks of age: 17.5% *Prop1* ^df/df^ and 19.5% *Prop1* ^-/-^ for each on N4 B6, p = 0.69 [19]. The N4 B6 background, however, increased the risk of lethality after weaning in homozygotes for both of the *Prop1* mutant alleles.

### *Prop1* deficient mice exhibit elevated levels of circulating ACTH and corticosterone

**Figure 1:**
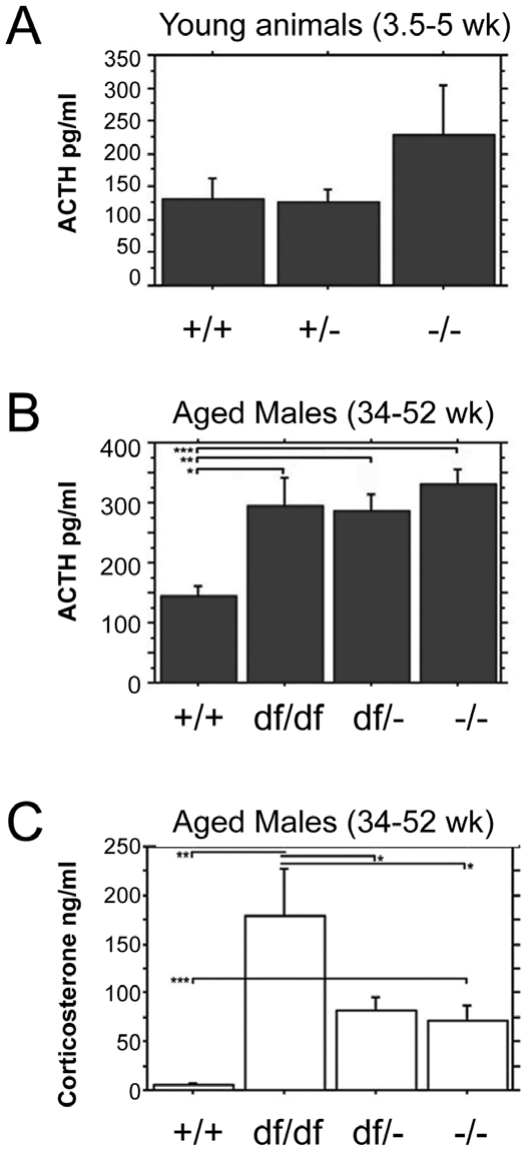
No evidence for evolving hypocortisolism in *Prop1* deficient animals. Blood plasma was collected from 3.5 to 5 wk N4B6 (Panel A) and 34 to 52 wk mixed genetic background (Panel B) animals from and the circulating ACTH levels were determined by RIA. Males and females were included together because the individual analysis showed no difference in the ACTH levels of aged-matched animals of the same genotype. At 3.5 to 5 weeks *Prop1^-/-^* (n = 6) animals tended to have higher circulating levels of ACTH than *Prop1^+/^* ^−^ (n = 10) or *Prop1^+/+^* (n = 10) animals, but the difference was not statistically significant (top). At 34 to 52 weeks three different genotypes of *Prop1* mutant animals, *Prop1* ^-/-^ (n = 8), *Prop1* ^df/-^ (n = 20), and *Prop1* ^df/df^ (n = 12), exhibited an increase in circulating ACTH levels compared to *Prop1^+/+^* (n = 9) (bottom). Values represent the mean ACTH production (pg/mL) ± SE. *, *P*<0.01; **, *P*<0.005; ***, *P*<0.0005. Corticosterone levels were measured in serum from aged male *Prop1^+/+^* (n = 4), *Prop1* ^df/df^ (n = 4), *Prop1* ^df/-^ (n = 11), and *Prop1* ^-/-^ (n = 7) mice (Panel C). All three genotypes of *Prop1* deficient mice show elevated basal levels of corticosterone compared to wild type. *Prop1* ^df/df^ mice have statistically higher basal levels of corticosterone compared to *Prop1* ^df/-^ or *Prop1* ^-/-^ mice. Values represent the mean corticosterone (ng/mL of blood) ± SE. *, *P*<0.005; **, *P*<0.0005; ***, *P*<0.05.

To determine whether the observed post-weaning lethality on the N4 B6 background could arise from evolving hypocortisolism, we examined ACTH and corticosterone production. We analyzed the serum of 3.5 to 5 week old *Prop1* ^-/-^ and normal mice on the N4 B6 background by RIA to address the ability of *Prop1* mutant corticotrophs to secrete ACTH (**Fig. 1**). There was no evidence for reduced ACTH production. Although these N4 B6-*Prop1* ^-/-^ mice showed a trend towards increased serum ACTH compared to wild type and heterozygote littermates, the difference was not significant. Western blots revealed similar ACTH protein content in the pituitary glands of normal and *Prop1* mutant mice (data not shown).

To determine whether *Prop1* mutants exhibit evolving hypocortisolism at older ages we aged *Prop1* mutant animals with three different genotypes and genetic backgrounds (*Prop1* ^-/-^, *Prop1* ^df/-^, and *Prop1* ^df/df^) to 7–12 months old and measured both ACTH and corticosterone. All genotype combinations of *Prop1* mutants had significantly elevated ACTH and corticosterone (**Fig. 1**). ACTH levels were 2 to 2.5x elevated in mutants relative to normal littermates, and the corticosterone levels were even more dramatically heightened in mutants. Our evidence for up regulation of the pituitary-adrenal axis in *Prop1* deficient mice is consistent with previous reports of elevated corticosterone in Ames dwarf mice [31], [32], and the increased corticosterone levels we reported in *Prop1* ^-/-^ newborns [19]. Thus, there is no evidence that *Prop1* mutant mice develop the age related ACTH deficiency and hypocortisolism that has been observed in some human patients with *PROP1* mutations.

### *Prop1* deficient mice respond to restraint stress

**Figure 2:**
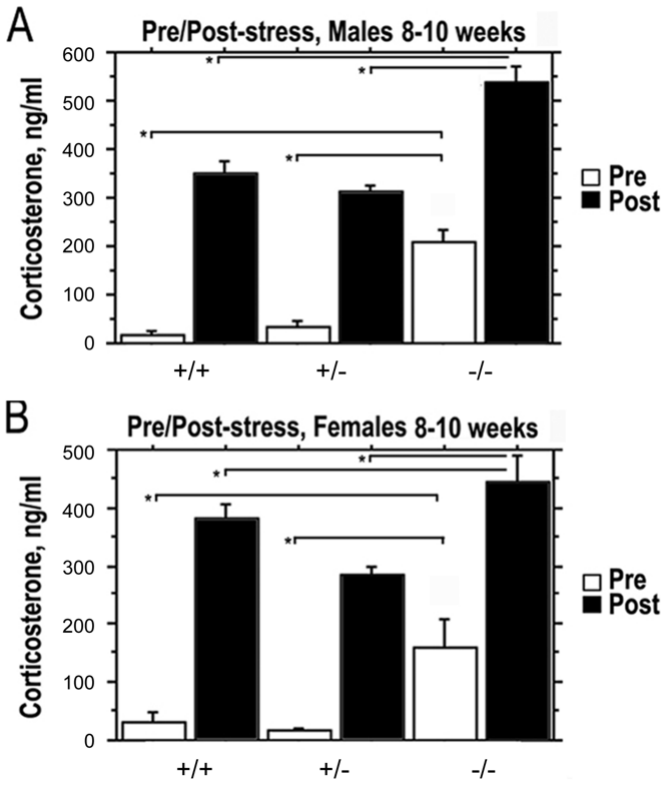
Elevated basal corticosterone levels in young adult *Prop1* deficient mice become higher in response to restraint stress. RIA analysis of circulating corticosterone was carried out on serum from 8 to 10 week males (A) and females (B) of segregating the *Prop1* null allele at N4 B6 prior to (white bars) and following restraint stress (black bars). Male *Prop1* ^-/-^ (n = 6) had significantly elevated basal and post-stress levels of corticosterone compared to *Prop1^+/−^* (n = 7) and *Prop1^+/+^* (n = 3). Values represent the mean corticosterone (ng/mL of blood) ± SE. *, *P*<0.0001. Female *Prop1^-/-^* (n = 3) mice had both elevated basal and post-stress levels of corticosterone compared to *Prop1^+/−^* (n = 5) and *Prop1^+/+^* (n = 6). Values represent the mean corticosterone (ng/mL of blood) ± SE. *, *P*<0.005.

Stress increases pituitary ACTH release and subsequent corticosterone secretion by the adrenal gland [27], [33], [34]. We exposed *Prop1* mutant and normal animals to restraint stress to test the ability their pituitary-adrenal axis to respond to this challenge (**Fig. 2**). Serum corticosterone levels were measured in N4 B6 *Prop1^-/-^*, *Prop1^+/^* ^−^ and *Prop1^+/+^* male and female mice at 8–10 weeks of age prior to and following 30 min of restraint stress. *Prop1* ^-/-^ animals had dramatically elevated basal, serum levels of corticosterone compared to wild type and *Prop1^+/^* ^−^ mice (**Fig. 2A and 2B**, white bars). Basal corticosterone was 4 fold higher in nonstressed male mutants than normal littermates, and 3 fold higher for female mutants vs. normals. Following restraint stress, both *Prop1^-/-^* males and females exhibited elevated serum corticosterone compared to *Prop1^+/-^* and wild type mice (**Fig. 2A and 2B** **,** black bars). The fold increase in corticosterone from basal to post-stress measurements is less for *Prop1* ^-/-^ animals compared to the wild type (2-3 fold compared to 11-16 fold). While this could be described as a blunted response, the absolute value of circulating corticosterone following restraint was higher in mutants than normal littermates. Post stress, the corticosterone values for male and female mutants were 504 +/− 29 ng/ml and normal littermates were 373 +/− 18 ng/ml. The post stress values in the 500 ng/ml range may be the maximal response. Thus, there is no evidence for impaired pituitary-adrenal axis function.

### *Prop1* mutant adrenal glands are enlarged relative to body weight

**Figure 3:**
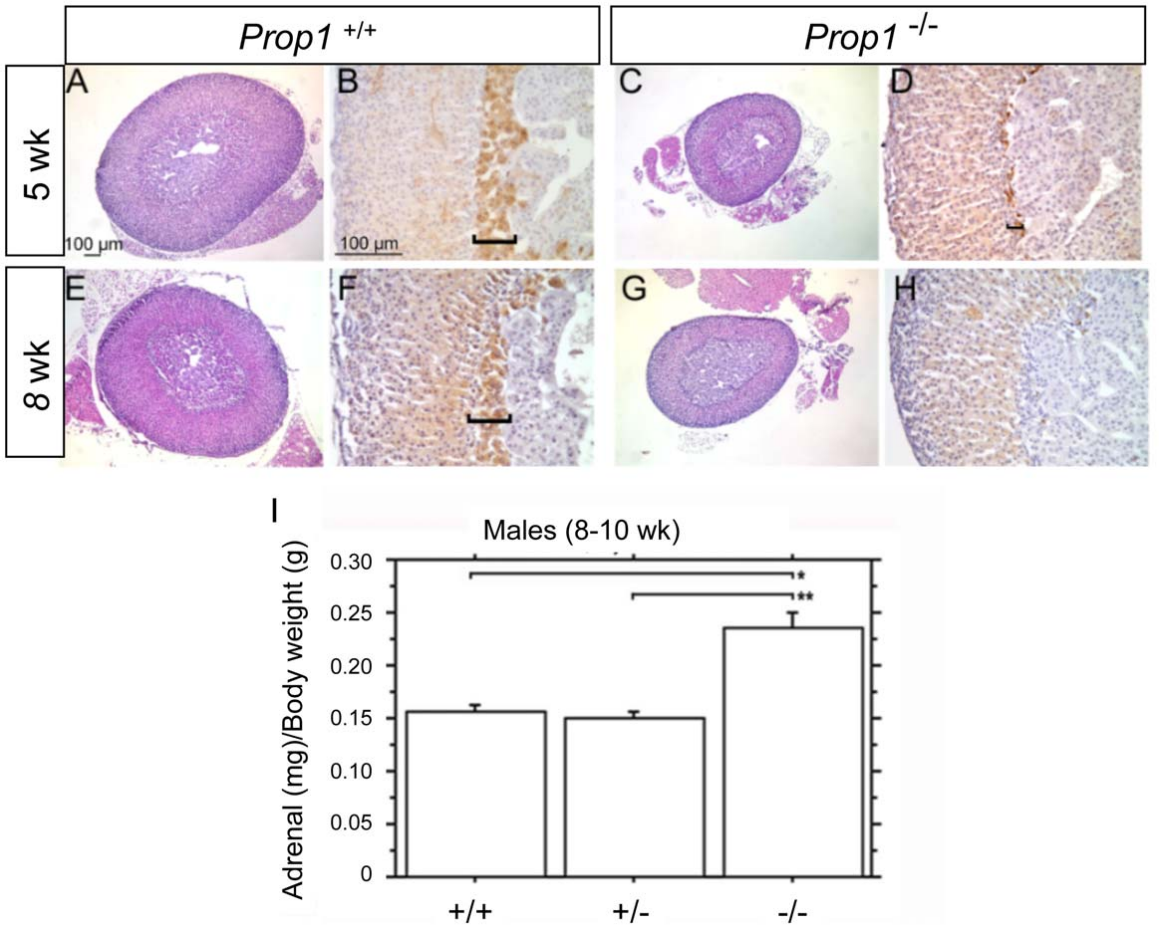
Adrenal glands of *Prop1* deficient mice are not hypotrophic. Adrenal glands were dissected from 5 and 8 week old female N4 B6 *Prop1^+/+^* and *Prop1^-/-^* mice, fixed, embedded, sectioned, and stained with hemotoxylin and eosin (Panels A, C, E, G) and immunostained for 20α-hydroxysteroid dehydrogenase [59] and developed with diaminobenzidine (brown, Panels B, D, F, H) to visualize the X-zone (brackets). The ratio of adrenal weight to body weight (Panel I) was increased in *Prop1* ^-/-^ (n = 5) compared to *Prop1^+/−^* (n = 6) or *Prop1^+/+^* (n = 3) N4 B6 male mice at 8 to 10 wks. Values represent the mean adrenal weight (mg) per body weight (g) ± SE. *, *P*<0.0001; **, *P*<0.0005.

ACTH is important for the development and growth of the adrenal gland in mice and other mammals [35], [36], [37]. The adrenal weights of N4 B6 *Prop^-/-^* mice were compared to *Prop1^+/−^* and wild type to determine the consequence of elevated ACTH on adrenal growth. The absolute size of the adrenal gland is smaller in the *Prop1* ^-/-^ dwarf males compared to wild type. However, the ratio of adrenal weight to body weight is actually increased in the *Prop1* ^-/-^ males compared to wild type (**Fig. 3**). This is consistent with the chronically elevated ACTH secretion in *Prop1^-/-^* mice.

The mouse adrenal gland is comprised of the adrenal medulla, which is important for the production of catecholamines such as norepinepherine and epinephrine, and the adrenal cortex which is important for corticosterone biosynthesis and contains the *zona glomerulosa* and *zona fasciculata* [38]. We examined adrenal development and morphology in N4 B6 normal and *Prop1* ^-/-^ male and female mice at 3.5, 5, 8, 10 weeks of age. The zona fasciculata and zona glomerulosa are morphologically indistinguishable in normal and mutant mice (**Fig. 3**, and data not shown). The adrenal X-zone is typically present between the *zona fasciculata* and medulla throughout postnatal development and then regresses in male mice starting at 3 weeks of age and in females during the first pregnancy [39]. The X-zone is not well understood, but it is thought to be analogous to the fetal zone in the human adrenal gland. Growth of the X-zone is regulated by pituitary gonadotropins and activin [40]. The X-zone is marked by 20α-hydroxysteroid dehydrogenase immunostaining and is present but smaller in female *Prop1* ^-/-^ mice at 5 wks and nearly undetectable at 8 wks [41] (**Fig. 3**). Thus, the X-zone is formed but is underdeveloped and apparently regresses early in *Prop1* mutants.

We used Western blotting to evaluate the levels of steroidogenic enzymes in *Prop1* ^-/-^ adrenals (data not shown). Similar levels of 21-hydroxylase enzyme, which is important for corticosterone biosynthesis [42], [43], steroidogenic acute regulator protein (StAR), which mediates the acute steroidogenic response [44] and the p450 cholesterol side chain cleavage protein (SSC) [45], [46], were observed in *Prop1* ^-/-^ adrenals compared to *Prop1^+/−^* or wild type. These results are consistent with functioning adrenal glands in *Prop1^-/-^* mice.

### *Prop1* deficiency causes low blood glucose

**Figure 4:**
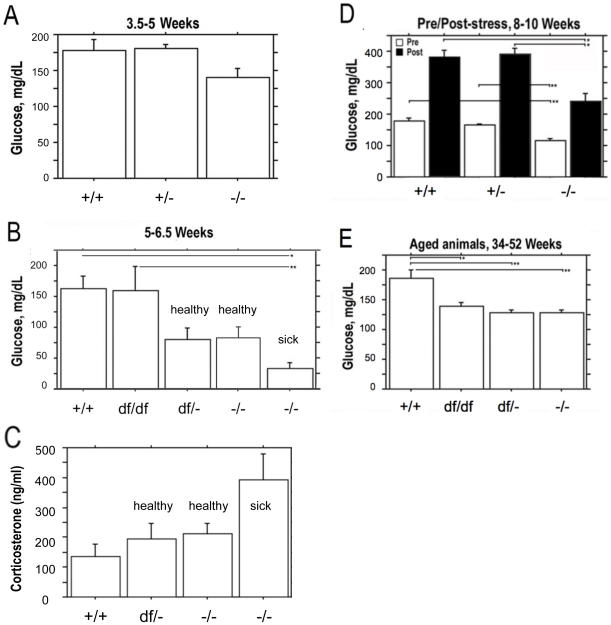
*Prop1*-defiency results in low blood glucose levels. Blood glucose levels were measured in normal and *Prop1* mutant mice at four ages. (A) Basal glucose levels in 3.5 to 5 week *Prop1^-/-^* mice (n = 6) were lower than *Prop1^+/+^* (n = 10) and *Prop1^+/−^* (n = 10) mice from mixed genetic backgrounds, but the difference was not statistically significant at this age. (B) On mixed genetic backgrounds the blood-glucose measurements from 5 to 6.5 wk old *Prop1^+/+^* (n = 6) and *Prop1* ^df/df^ (n = 6) were normal, but *Prop1* ^df/-^ (n = 3), *Prop1^-/-^* healthy (n = 12) and *Prop1^-/-^* wasting (n = 7) mice had significantly decreased blood-glucose levels. Values represent the mean blood glucose levels (mg glucose/dL blood) ± SE. *, *P*<0.01; **, *P*<0.005. (C) The low glucose levels in mutants shown in panel B are associated with elevated corticosterone levels (ng corticosterone/ml blood +/− SE.) (D) Blood-glucose levels were measured in 8 to 10 week old mice of the N4 B6 background prior to (white bars) and following restraint stress (black bars). *Prop1^-/-^* (n = 8) mice had decreased basal and post-stress blood-glucose levels compared to *Prop1^+/+^* (n = 9) and *Prop1^+/−^* (n = 11). Values represent the mean blood glucose levels (mg glucose/dL blood) ± SE. *, *P*<0.0001; **, *P*<0.0005. (E) Blood-glucose levels in 34 to 52 wk old mice on mixed genetic background were decreased in all genotypes of *Prop1* mutants, *Prop1^df/df^* (n = 4), *Prop1^df/-^* (n = 11), *Prop1^-/-^* (n = 7), compared to normals, *Prop1^+/+^* (n = 4). Values represent the mean blood glucose levels (mg glucose/dL blood) ± SE. *, *P*<0.005; **, *P*<0.0005; ***, *P*<0.0001.

We hypothesized that reduced glucose levels secondary to growth hormone deficiency could cause the elevated basal levels of ACTH and corticosterone in the blood of *Prop1* deficient mice. *Prop1* deficient mice produce very few somatotrophs and lack detectable circulating GH [18], [47]. GH has pleiotropic functions that involve many target organs. In the liver GH activates the production of insulin-like growth factor 1 (*Igf1*) [48]. Quantitative RT-PCR measurements revealed a 50-fold decrease in *Igf1* expression in the *Prop1* ^-/-^ mouse livers compared to wild type (data not shown). Growth hormone is important for metabolism and glucose homeostasis though its role in modulating *Igf1* production [49]. GH deficiency can cause hypoglycemia in rodents and humans [50]. We performed blood glucose measurements on a variety of different *Prop1* mutant genotypes at several ages (**Fig. 4**). At 3.5 to 5 wks the blood-glucose level of *Prop1* ^-/-^ mice (N4 B6 background) is similar to that of heterozygous littermates and wild types, 140 +/− 14 mg/dL vs. 177 +/− 16 mg/dL, p = 0.048 (**Fig. 4A**). By 5 to 6.5 weeks however, the N4 B6 *Prop1* ^-/-^ mice had approximately two-fold lower blood-glucose levels than either *Prop1* ^+/+^ or *Prop1^df^* ^/+^ mice, 80 +/− 18 vs. 162 +/− 22 mg/dL, respectively (**Fig. 4B**). Thus, mutants this age have borderline hypoglycemia since a level of less than 60 mg/dL is considered clinically hypoglycemic. Mice affected by wasting were clearly hypoglycemic with blood glucose at 36 +/− 9 mg/dL (**Fig. 4B**). Moreover, the corticosterone levels in wasting mice 5 to 6.5 wk old mice are strikingly elevated: 2.9 fold relative to wild type and 1.9 fold relative to healthy mutants (**Fig. 4C**). The corticosterone values are 136 +/− 40 in *Prop1* ^+/+^ (N = 11), 193 +/− 55 in *Prop1* ^df/-^ (N = 12), and 211 +/− 36 in healthy *Prop1* ^-/-^ (N = 12), and 393 +/− 86 in sick *Prop1* ^-/-^ mice, (N = 7). The very high corticosterone levels support the idea that the wasting phenotype is not due to failure of the pituitary adrenal axis. The elevated levels are consistent with a response to metabolic stress, but it is difficult to determine whether the cachexia is the cause or the effect of severe hypoglycemia.

The low glucose levels persist in older *Prop1* deficiency mice. At 8–10 weeks the N4 B6 *Prop1* ^-/-^ mice had lower glucose levels (ave. 117 +/− 4 mg/dL) than controls (178 +/− 10 mg/dL) (**Fig. 4D**). All genotype combinations of *Prop1* mutants had reduced serum glucose levels at older ages, 34–52 weeks, although the levels were not low enough to be considered clinically hypoglycemic: 186 +/− 14 mg/dL for *Prop1^+/+^*, 139 +/− 6 *Prop1* ^df/df^, 128 +/− 5 *Prop1* ^df/-^, and 128 +/− 4 mg/dL for *Prop1* ^-/-^ (**Fig. 4E**). Thus, all genotype combinations of mutants have significantly lower glucose levels after 5 wks (p<0.0001), with the lowest levels in wasting mice.

We tested whether N4 B6 *Prop1* ^-/-^ mutants would respond to 30 min restraint stress with elevated glucose levels (**Fig. 4D**). The pre- and post-stress values for mutants were 117 +/− 4 and 242 +/− 23 mg/dL, and the pre- and post-stress control values were 178 +/− 10 and 382 +/− 22. Although the mutants responded with elevated blood glucose, their post-stress glucose levels were lower than control littermates. The fold change pre- and post-stress, however, was similar in mutants and normal littermates. These results demonstrate that *Prop1* deficiency causes a reduction in circulating glucose levels, but this deficiency does not block the elevation of blood glucose in response to stress.

## Discussion

The main goal of this research was to study the pituitary-adrenal axis in two different mutant *Prop1* alleles on different genetic backgrounds to detect any evidence of ACTH deficiency and subsequent hypocortisolism. If ACTH deficiency were detected, then the mice would correspond to the findings of acquired hypocortisolism in human MPHD patients with lesions in the *PROP1* gene [11], [12], [13]. We found no evidence for reduced pituitary-adrenal axis function in *Prop1* mutant mice. Instead, the pituitary adrenal axis is activated, including both elevated ACTH and corticosterone in the setting of blood low glucose levels. These results are consistent with reports for DF/B-*Prop1^df/df^* mice [31]. The GH deficiency of *Prop1* mutant mice is associated with reduced transcription of *Igf1* in the liver, reduced blood glucose levels, and activation of the pituitary adrenal axis. Despite these metabolic alterations, affected mice are able to mount a stress response yielding further elevations of ACTH, glucocorticoids, and circulating glucose. Thus, we find no evidence of impaired pituitary-adrenal axis function in *Prop1* deficient mice for either the df or null alleles on the backgrounds and ages tested. While we cannot rule out the possibility that some combination of parameters could provoke hypocortisolism in *Prop1* mutant mice [51], it appears that evolving ACTH deficiency is a feature that distinguishes mutant mice from the human patients with *PROP1* mutations.

Both *Prop1* null and df mutant mice have the lowest circulating glucose levels of 25-75 mg/dl between weaning and adulthood, which is sometimes associated with lethality of unknown cause. We observed the highest susceptibility to lethality after 5 wks on the B6 strain background, irrespective of the *Prop1* mutant allele. Normal B6 mice have a lower body weight and food intake than many other strains during the time when *Prop1* mutant lethality occurs (Jax phenome database; http://www.jax.org/phenome). It is possible that severe hypoglycemia contributes to the increased susceptibility of *Prop1* mutants to lethality on the B6 background, although other differences in metabolism may be responsible. For example, the livers of healthy *Prop1* deficient mice resemble livers of normal fasted mice, and sickly mutant livers are more affected (data not shown) [52]. The *Prop1* mutants that survive to adulthood have significantly longer life spans than their normal littermates, like other strains with reduced insulin like growth factor activity [32].

The lower glucose levels we observed in *Prop1* deficient mice are consistent with clinical data from human patients with GH deficiency. Approximately 5% of humans with GH deficiency also had hypoglycemia, and 10% of the hypoglycemic patients died [53]. Another study showed that approximately 3% (37/1366) of GH deficient children died and that 24% (9/37) of those who died suffered from severe hypoglycemia [54]. Pituitary aplasia also causes severe hypoglycemia, thus representing a serious life threatening problem in neonates with MPHD if not quickly treated [55], [56]. Differences in the GH signaling pathway involving AKT2 can cause hypoglycemia, seizures and death [57], [58]. The reason for the individual variation in susceptibility to severe hypoglycemia and lethality in humans and mice are not known.

We found no evidence for disruption of the pituitary-adrenal axis in *Prop1* deficient mice. In direct contrast to the human MPHD cases with progressive ACTH loss and hypocortisolism, *Prop1* deficient mice exhibit elevated ACTH and corticosterone and reduced glucose levels at 6 mo and 1 yr of age. Young adult *Prop1* deficient mice respond to restraint stress with further elevation of ACTH, corticosterone and glucose levels, and show no reduction in adrenal content of steroidogenic enzymes, indicating that the pituitary-adrenal axis can react functionally to this challenge. In addition, the adrenals of the *Prop1* ^-/-^ mice are enlarged relative to normal mice when normalized to body weight, as expected for chronic ACTH secretion in rodents and other mammals, including primates [36], [37]. Finally, sickly, young *Prop1* mutants have even higher corticosterone levels than healthy mutants.

The basis for the evolving nature of the hormone deficiencies, including hypocortisolism, in human *PROP1* deficient patients remains elusive. It is tempting to speculate that it arises from depletion of progenitors, but species differences in function are also possible. Genetic background affects the viability of young *Prop1* deficient mice, largely due to different responses of target organs to pituitary hormone deficiency. Multiple *Prop1* mutant alleles and genetic backgrounds support elevated ACTH and corticosterone levels and lower glucose levels that persist with age. Although mice with MPHD have been invaluable for understanding the molecular basis for human disorders of hormone-deficiency and dwarfism, pituitary growth, and pituitary cell specification, they may be less pertinent for understanding the nature of progressive hormone deficiency that characterizes humans with *PROP1* mutations.
